# *In Vitro* and *In Vivo* Profile of PPL-101 and PPL-103: Mixed Opioid Partial Agonist Analgesics with Low Abuse Potential

**DOI:** 10.3389/fpsyt.2017.00052

**Published:** 2017-04-12

**Authors:** Taline V. Khroyan, Andrea Cippitelli, Nicholas Toll, John A. Lawson, William Crossman, Willma E. Polgar, Lawrence Toll

**Affiliations:** ^1^SRI International, Menlo Park, CA, USA; ^2^Torrey Pines Institute for Molecular Studies, Port St. Lucie, FL, USA; ^3^Phoenix PharmaLabs Inc., Woods Cross, UT, USA

**Keywords:** analgesic, non-addicting, kappa opiate, self-administration, conditioned place preference

## Abstract

Opiates are still the most effective and widely used treatments for acute and chronic pain. However, the problems associated with morphine and other standard opioid analgesics severely limit their effectiveness in the clinic. PPL-101 and PPL-103 derived from morphine and morphinan ring systems contain a chiral N-substituent, which confers it with a unique combination of high-binding affinities and partial agonist activities at mu, delta, and kappa opioid receptors, leading to unique *in vivo* pharmacology compared to other conventional opioids. Acute antinociceptive and reward acquisition of PPL-101 and PPL-103 were assessed in mice using the tail flick assay and conditioned place preference (CPP) paradigm, respectively. The reinforcing effects of these compounds were assessed in rats using the self-administration paradigm. In mice, PPL-101 and PPL-103 produced antinociception reaching maximal effects that were equivalent to morphine at approximately 1/3 and 1/10 of morphine’s dose, respectively. PPL-101-induced antinociception was attenuated following pretreatment with the kappa antagonist JDTic, but not the mu opioid antagonist beta-FNA. In mice, PPL-101 and PPL-103 produced dose-dependent decreases in activity, similar to other kappa agonists; however, they did not produce conditioned place aversion, and in fact elicited a trend toward CPP. In rats, neither PPL-101 nor PPL-103 were self-administered when substituted for morphine and PPL-101 attenuated morphine self-administration, when administered systemically prior to the self-administration session. Collectively, these results indicate that mixed opioid receptor partial agonists can produce potent antinociceptive activity with a lack of aversion in mice and without being self-administered in rats. Compounds with this profile could be superior analgesics with greatly reduced addiction liability and fewer side-effects compared to traditional opiates.

## Introduction

It is well known that activation of the different opioid receptors induces different pharmacological actions. Mu receptor activation induces antinociception, along with a decrease in respiration and gut motility, and significant abuse liability (1, 2). Kappa receptor activation also induces antinociception but with reduced respiratory depression and reduced inhibition of gut motility (2–4). With respect to potential for abuse, kappa receptor agonists often induce dysphoria and accordingly are generally considered not to have abuse liability (5, 6). The ramifications of delta receptor activation are less clear. Although activation of delta receptors produces antinociception, the maximal effects are much less than that produced by mu receptor activation, and it appears to be primarily spinally mediated. However, it has been shown that delta antagonists may prevent mu-mediated tolerance development (7–9). In regard to reward/reinforcement, the findings with delta agonists are mixed, with some evidence pointing to delta-mediated reward, while other evidence suggests that even selective delta agonists require mu receptors to generate reinforcing properties (10–12).

The modification of opioid ligands leading to changes in efficacy, and receptor selectivity is fairly well established (13). Numerous studies have identified alternate ring structures. Morphinan and benzomorphan structures tend to mimic the pharmacological properties of morphine analogs with roughly similar potencies and consistent changes with respect to N-substituent modifications. Modification of the N-substituent on morphine, morphinans, and benzomorphans leads to changes in the binding affinity, receptor selectivity, and efficacy of the derivatives (14, 15). Substituting *N*-methyl with *N*-allyl or *N*-cyclopropylmethyl increases affinity for kappa and delta receptors without decreasing affinity at mu receptors (16), but it decreases the efficacy at mu receptors, leading to the development of opiate antagonists or partial agonists, such as naloxone, naltrexone, or cyclazocine. Thebaine-based opioids, such as etorphine and buprenorphine, wherein various alkyl groups are attached to thebaine through a diels-alder reaction, now have an additional ring. This modification often produces compounds with high affinity at each opioid receptor regardless of the N-substituent (16). Likewise, in flexible opiates without fused rings, such as fentanyls or phenylpiperidines, the N-substituent does not appear to modulate receptor affinity or efficacy (17).

Some compounds, such as nalorphine (*N*-allyl-normorphine), have antagonist activity at mu receptors but agonist activity at kappa, resulting in an analgesic compound without reward, but with dysphoria (18–20). The resulting dysphoria in humans makes nalorphine and similar compounds impossible to use as analgesics (21). A great deal of time and effort has been spent in trying to identify the opioid “Holy Grail” with the most beneficial N-substituent, which can result in a compound with potent antinociceptive activity and reduced euphoric effects, but without kappa-mediated dysphoria. In theory, attenuation in rewarding properties can be attained with a compound that has reduced efficacy at mu receptors, without activation of kappa receptors. An example of this type of compound would be buprenorphine, which is a mu partial agonist and kappa antagonist and has reduced addiction liability compared to a full agonist such as morphine (22, 23). Nevertheless, buprenorphine is increasingly being abused, which is why it is now being mixed with naloxone in the opioid addiction treatment medication Suboxone. Another profile that might meet these criteria could be a full/partial mu receptor agonist with kappa agonist activity, such as that found in pentazocine, nalbuphine, and butorphanol. These clinically used drugs have reduced abuse liability but with some problems with dysphoria in some patients and precipitation of withdrawal in opioid-addicted patients (20, 24, 25). An additional potential profile for a non-addicting analgesic without dysphoria could be a partial kappa agonist with some mu activity in order to moderate and titrate rewarding versus dysphoric aspects of the compound. Such compounds are exemplified by PPL-101 and PPL-103.

PPL-101 (α-methyl-cyclopropylmethyl-morphine) and PPL-103 (α-methyl-cyclopropylmethyl-morphinan) are opiate derivatives that bind with high affinity to mu and kappa receptors, with slightly lower affinity to delta receptors (Table 1). The α-methyl constituent produces two diasteriomers and constrains the cyclopropylmethyl moiety into an R or S orientation. Previous studies have demonstrated that the R orientation has higher affinity for the opiate receptors (26). Studies described here along with those conducted previously by the Committee for Problems on Drug Dependence (27, 28) demonstrate that PPL-101 and PPL-103 have unusual profiles and these, or similar compounds, could be ideal as analgesics with low abuse potential or potentially as drug abuse medications.

## Materials and Methods

### Animals

For assessment of antinociception and conditioned place preference (CPP), male ICR mice weighing 25–30 g at the start of the experiment were used. Mice were group-housed under standard laboratory conditions using nestlets as environmental enrichment in their cages and were kept on a 12:12-h day/night cycle (lights on at 7:00 a.m.). For experiments examining antinociception, animals were housed 10/cage and for the CPP experiments animals were housed 4/cage. Testing was conducted during the animals’ light cycle between 9 a.m. and 2 p.m. Animals were handled for 3–4 days before the experiments were conducted. On behavioral test days, animals were transported to the testing room and acclimated to the environment for 1 h.

For self-administration experiments, male Sprague-Dawley rats (200–225 g) were obtained from Charles River (Portage, MI, USA). Rats were housed in a room with a reverse 12-h light/12-h dark cycle (lights off at 7:30 a.m.). All self-administration experiments were conducted during the dark phase of the cycle. Animals were acclimated for 7 days with water and chow (Teklad Diets, Madison, WI, USA) and handled for 3 days before the experiments were conducted. Throughout all operant procedures, rats were food restricted and received 16–20 g of chow daily, to maintain 80% of free-feeding weight. Water was freely accessible.

### Drugs

**Figure 1:**
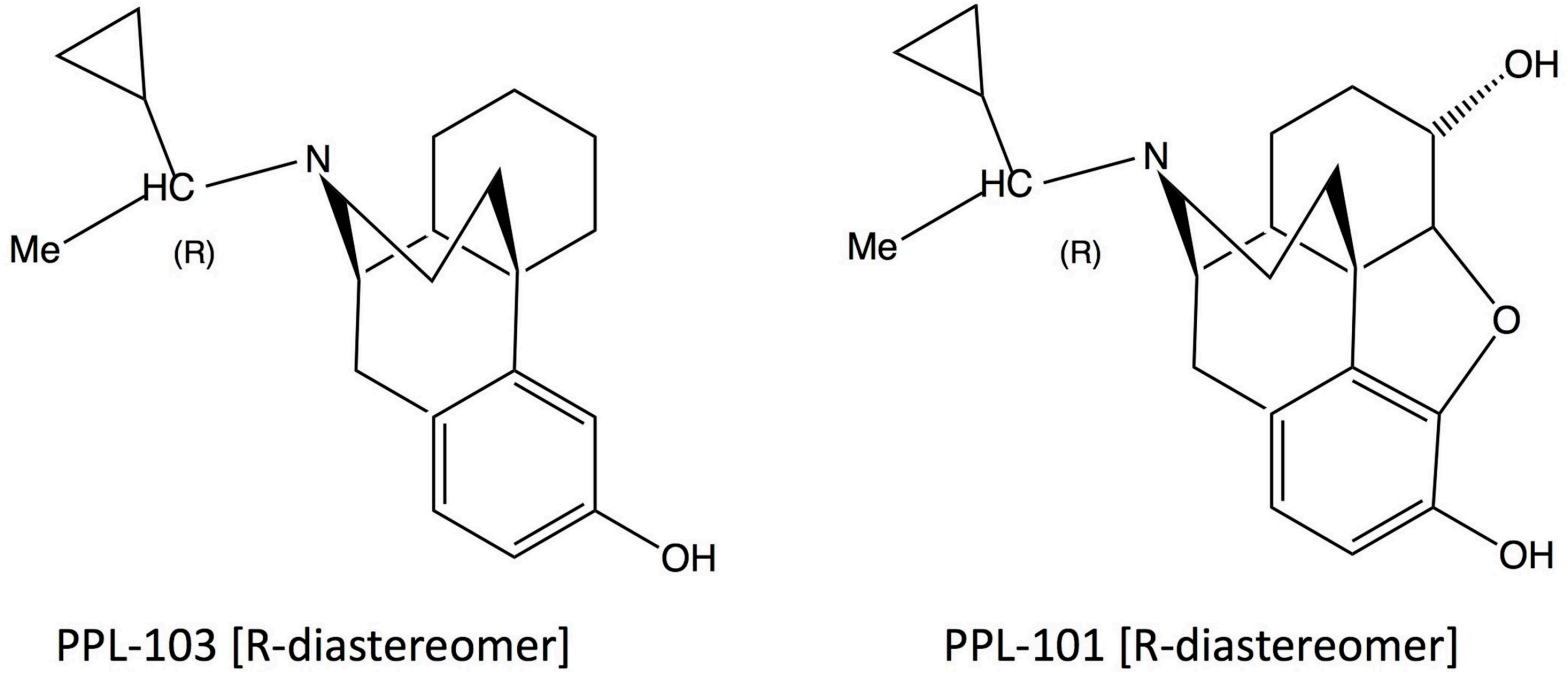
**Structures of PPL-103 and PPL-101**.

PPL-101 (α-methyl-cyclopropylmethyl-morphine) and PPL-103 (α-methyl-cyclopropylmethyl-morphinan) (Figure 1) were synthesized as described previously and used as the hydrochloride salt (29, 30). Morphine sulfate, beta-FNA, and the kappa agonist U-69,593 were provided by the National Institute of Drug Abuse, whereas JDTic was obtained from Dr. Ivy Carroll.

For studies in mice, PPL-101 and PPL-103 were dissolved in 3% DMSO and 0.5% aqueous hydroxypropylcellulose, whereas morphine hydrochloride, beta-FNA, and JDTic were dissolved in distilled water. All drugs except beta-FNA were injected in a volume of 0.1 ml/30 g subcutaneous (s.c.) Beta-FNA was made up as a 20 mg/kg solution and injected in a volume of 0.2 ml/30 g, s.c. to get the desired dose of 40 mg/kg. Controls received the appropriate volume of vehicle (i.e., 0.1 or 0.2 ml/30 g depending on the experiment). Doses of morphine, PPL-101, PPL-103, JDTic, and beta-FNA are reported as the salt.

For studies in rats, intravenous (i.v.) solutions of morphine, PPL-101, and PPL-103 were produced by dissolving drugs in 0.9% saline and then adjusted to pH 7.0–7.4 with 3 M sodium hydroxide. Morphine, PPL-101, and PPL-103 self-administration doses are reported as free base concentrations. Systemic solutions of PPL-101 and naloxone were made by dissolving drugs in 0.9% saline and were administered in a volume of 1 ml/kg, intraperitoneal (i.p.) JDTic was dissolved in distilled water and administered *via* the i.p. route. The kappa agonist U-69,593 was suspended in 10% DMSO, 10% TWEEN 80, and 80% distilled water and given s.c. 15 min prior to the plantar test.

### *In Vitro* Characterization

#### Cell Culture

All receptors were individually expressed in CHO cells stably transfected with human receptor cDNA, The cells were grown in Dulbecco’s modified Eagle medium with 10% fetal bovine serum, in the presence of 0.4 mg/ml G418 and 0.1% penicillin/streptomycin, in 100-mm polystyrene culture dishes. For binding assays, the cells were scraped off the plate at confluence. Receptor expression levels were 1.6, 1.8, and 3.7 pmol/mg protein for the mu, kappa, and delta opioid receptors respectively.

#### Receptor Binding

Binding to cell membranes was conducted in a 96-well format, as described previously (16, 31). Briefly, cells were removed from the plates, homogenized in 50 mM Tris pH 7.5, using a Polytron homogenizer, then centrifuged once, and washed by an additional centrifugation at 27,000 × *g* for 15 min. The final pellet was resuspended in Tris, and the suspension incubated with [^3^H]DAMGO (51 Ci/mmol, 1.6 nM), [^3^H]Cl-DPDPE (42 Ci/mmol, 1.4 nM), or [^3^H]U-69,593 (41.7 Ci/mmol, 1.9 nM) for binding to mu, delta, and kappa receptors, respectively. Non-specific binding was determined with 1 µM of unlabeled DAMGO ([D-Ala2, N-MePhe4, Gly-ol]-enkephalin), DPDPE ([D-Pen2,D-Pen5]Enkephalin), and ethylketocyclazocine (EKC), respectively. Samples were incubated for 60 min at 25°C in a total volume of 1.0 ml, with 15 µg protein per well. The reaction was terminated by filtration using a Tomtec 96 harvester (Orange, CT, USA) through glass fiber filters and radioactivity was counted on a Pharmacia Biotech beta-plate liquid scintillation counter (Piscataway, NJ, USA). IC_50_ values were calculated using Graphpad/Prism (ISI, San Diego, CA, USA), and *K*_i_ values were determined by the method of Cheng and Prusoff (32).

#### [^35^S]GTPγS Binding

[^35^S]GTPγS binding was conducted as described previously (33). Briefly, cells were scraped from tissue culture dishes into 20 mM Hepes, 1 mM EDTA, then centrifuged at 500 × *g* for 10 min. Cells were re-suspended in this buffer and homogenized using a Polytron Homogenizer. The homogenate was centrifuged at 27,000 × *g* for 15 min, and the pellet re-suspended in Buffer A, containing: 20 mM Hepes, 10 mM MgCl_2_, 100 mM NaCl, pH 7.4. The suspension was re-centrifuged at 27,000 × *g* and suspended once more in Buffer A. For the binding assay, membranes (8–15 µg protein) were incubated with [^35^S]GTPγS (50 pM), GDP (10 µM), and the appropriate compound, in a total volume of 1.0 ml for 60 min at 25°C. Samples were filtered over glass fiber filters and counted as described for the binding assays. Statistical analysis was conducted using the program Prism.

### *In Vivo* Studies

#### Assessment of Thermal Nociception of PPL-101 and PPL-103 in Mice Using the Tail Flick Assay

Acute nociception was assessed using the tail flick assay with an analgesia instrument (Stoelting) that uses radiant heat. This instrument is equipped with an automatic quantification of tail flick latency, and a 15 s cutoff to prevent damage to the animal’s tail. During testing, the focused beam of light was applied to the lower half of the animal’s tail, and tail flick latency was recorded. Baseline values for tail flick latency were determined before drug administration in each animal. The mean basal tail flick latency was 4.83 ± 0.07 SEM.

In initial experiments using both PPL101 and PPL103, following baseline measures, animals (*n* = 8 group) received a s.c. injection of their assigned dose of PPL-101 (0.3–10 mg/kg, s.c.) or PPL-103 (0.3–3.0 mg/kg, s.c.) and were tested for tail flick latencies 1-h post-injection. In follow-up experiments, time course of PPL-101-induced antinociception was examined looking at tail flick latency at 0.5-, 1-, 2-, and 4-h post-injection. In all of these experiments, separate groups of animals served as vehicle controls and received an injection of vehicle prior to testing, whereas positive controls received 15 mg/kg morphine prior to testing. The experimenters were blinded to the treatment and condition of each animal.

To determine the relative contributions of mu and kappa activity to the observed antinociceptive effects of PPL-101, separate groups of animals (*N* = 8/group) were pre-treated with mu opioid antagonist beta-FNA (40 mg/kg, s.c.) or kappa opioid antagonist JDTic (10 mg/kg, s.c.). In these experiments, PPL-101 was given 24-h following antagonist pretreatment and tail flick latencies were measured at 0.5-, 1-, 2-, and 4-h post-PPL-101 administration. Separate groups of animals (*N* = 8/group) received vehicle injections 24 h following pretreatment with beta-FNA or JDTic. In addition, there was a control group that received two vehicle injections 24 h apart (*N* = 9). Doses and pretreatment time of the antagonists were chosen based on previous research (34, 35).

#### Assessment of Reward Acquisition and Global Activity of PPL-101 and PPL-103 Using the CPP Paradigm in Mice

##### CPP Apparatus

The apparatus consisted of rectangular Plexiglas chambers divided into two distinct equal-sized compartments (19 cm × 22.8 cm × 18 cm high, Lafayette Instruments). One compartment had cedar-scented bedding underneath a bar grid floor and all but the front walls were black. The other compartment had pine-scented bedding beneath a mesh floor and all but the front wall were white. The front walls were transparent so that the animal’s behavior could be monitored. A removable partition divided the two compartments. During conditioning, the compartments were divided by a solid partition. On the CPP test day, the solid partition was replaced with a partition that had an opening, allowing the animal free access to both compartments. A video camera that was linked to a computer was mounted above the chambers and tracked the animals’ movement. Previous experiments using this setup have indicated that the apparatus is unbiased, as untreated animals do not show a preference for one compartment over the other.

##### Conditioning Training

Each conditioning trial was composed of two sessions conducted over two consecutive days. During the drug session, animals received a s.c. injection of their respective dose of PPL-101 (1.0–3.0 mg/kg, *N* = 6–8/group) or PPL-103 (0.3–3.0 mg/kg, *N* = 8/group) and were confined to one of the compartments for 30 min. On a separate day, the vehicle session, animals received an injection of vehicle and were confined to the alternate compartment for 30 min. Each 2-day conditioning trial was repeated over six consecutive days (three trials) such that animals received three drug sessions and three vehicle sessions. The particular compartment paired with the drug and the order of placement into the drug-paired versus saline-paired compartment was counterbalanced across groups. A group of mice received vehicle in both compartments and served as controls (*N* = 8). The positive control group received morphine (15 mg/kg; *N* = 8) during their drug session.

##### CPP Test Day

Twenty-four hours after the last conditioning session, the animals were given access to both compartments simultaneously for 15 min and the amount of time that animals spent in each compartment was recorded.

##### Acute and Repeated Measures of Global Activity

During conditioning, overall global activity of the animals after acute and repeated drug injection was also recorded. These data were captured by the spontaneous motor recording and tracking software system (Panlab), a color image capturing system that works in real time and tracks all the movements of the animal, for a given amount of time *via* a video camera connected to the computer. Given that this system tracks all movement and records this gross measure we have termed this global activity since it encompasses fine movement, movement due to rearing, grooming, sniffing, and locomotor activity.

#### Assessment of Reinforcing Effects of PPL-101 and PPL-103 Using Self-administration in Rats

##### Apparatus

Self-administration experiment were conducted in operant chambers (Med Associates, Inc., St. Albans, VT, USA) enclosed in lit, sound attenuating, ventilated environmental cubicles. Each chamber was equipped with two retractable levers located in the front panel and a food pellet magazine was located between the two levers. A pellet dispenser was positioned behind the front panel of the boxes. Chambers were also equipped with auditory stimuli presented *via* a speaker and visual stimuli located above the levers (cue lights). Infusions occurred by means of syringe pumps (Med Associates, Inc., St. Albans, VT, USA) and liquid swivels (Instech Solomon, Plymouth Meeting, PA, USA), connected to plastic tubing protected by a flexible metal sheath for attachment to the external catheter terminus. During self-administration, an infusion pump was activated by responses on the right (active) lever, while responses on the left (inactive) lever were recorded but did not result in any programmed consequences. Activation of the pump resulted in a delivery of 0.1 ml of the fluid. A microcomputer controlled the delivery of reinforcers, presentation of auditory and visual stimuli, and recording of the behavioral data.

##### Food Training

One week after arrival, all rats were trained for 3 days to lever-press for 45 mg food pellets (Test Diet, 5-TUM, Richmond, IN, USA) under a fixed ratio 1 (FR-1) schedule of reinforcement during 30-min sessions.

##### i.v. Catheterization

Intravenous catheterization was performed after operant food training and under inhalation of isoflurane anesthesia as previously described (36, 37). To maintain patency for the duration of the experiment, catheters were flushed daily with 0.2 ml of heparinized (1,000 UPS U/ml) saline solution containing enrofloxacin (0.7 mg/ml).

##### Morphine Self-administration Training

Following recovery from surgery, all animals were trained to self-administer morphine (100 µg/kg/infusion) in the same chambers as the food training sessions, using an FR-1 [20 s time out (TO)] schedule during daily 2-h sessions (38) conducted 5 days/week. This initial training lasted 7 days. TO was concurrent with illumination of a cue light located above the active lever to signal delivery of the positive reinforcement. An intermittent tone (7 kHz and 70 dB) was sounded throughout the session. Responses to the inactive lever were recorded and served as a measure of non-specific motor behavior.

##### Assessment of the Reinforcing Effects of PPL-101

Following 1 week of morphine self-administration using FR1TO20, rats (*N* = 24) were divided in four groups of *N* = 6, so that the average morphine self-administration rate based on their lever pressing performance on the last 3 days of morphine training was the same across all groups. To examine whether PPL-101 would substitute for morphine, two groups of rats were given access to PPL-101 (30 and 100 µg/kg/infusion, respectively), while the third group was given access to vehicle (0.9% saline), and the fourth group continued morphine (100 µg/kg/infusion) self-administration. This self-administration period was conducted under the FR1TO20 task and lasted for an additional seven sessions (testing over 5 days/week). Results are reported as number of infusions earned in 120 min. After a week on this reinforcement schedule, the task was switched to progressive ratio (PR) schedule that better measures motivational properties rather than rate of drug intake (39). For the PR procedure, the response requirement for successive injections was 1, 2, 4, 6, 9, 12, 15, 20, 25, 32, 40, 50, 62, 77, 95, etc. (38), as derived from the formula “Response ratio (rounded to nearest integer) = [5e^(0.20 × inj. number)^] − 5” (40). The PR schedule used a 20-s TO following each drug infusion. Testing on this day was conducted until the animals reached the break point, defined as the highest ratio completed prior to a 60-min period during which no injections were earned and lasted a maximum of 4 h. Data are reported as number of infusions obtained during the PR session. On the following day, morphine self-administration (100 µg/kg/infusion) under FR1TO20 task was re-established in all rat groups. To carry out the subsequent experiments, the same cohort of rats was used except where otherwise specified.

##### Assessment of PPL-103 Reinforcing Effects

To examine whether PPL-103 would substitute for morphine, six 2-h sessions of morphine self-administration were initially conducted in a new cohort of (*N* = 8) rats using FR1TO20 schedule. Then, rats were given access to PPL-103 (0, 30, and 100 µg/kg/infusion) according to a Latin square within-subject design, in which each dose was tested 4 days/week under the FR1TO20 task and under PR on the fifth day. During the following weeks, rats had access to a different PPL-103 dose. Results of FR sessions are reported as number of infusions earned in 120 min. Total responses and break point were the dependent variable for PR.

##### Effect of Systemically Administered PPL-101 on Morphine Self-administration

After restoring a new morphine self-administration baseline of responding, animals (*N* = 8) were used to assess the effectiveness of systemically administered PPL-101 in altering morphine lever pressing. PPL-101 (0, 0.3, 1.0, and 3.0 mg/kg, i.p.) was tested using a Latin-square counterbalanced within-subjects design. Following the establishment of a stable morphine self-administration, for the test session, animals were treated with the desired dose of PPL-101 (i.p.) 15 min prior to the self-administration session. Following each test session day, animals were allowed 1 day off, and a new baseline of morphine self-administration was then established over the following 2 days, prior to the subsequent test session. Data are reported as number of infusions obtained in the 120-min test session. Responses on the inactive lever were also recorded and served as an index of unspecific motor behavior.

##### Effect of JDTic on Morphine and PPL-101 Self-administration

Two additional groups were trained to self-administer morphine (100 µg/kg/infusion; *N* = 5) or PPL-101 (100 µg/kg/infusion; *N* = 7) for 1 week under the FR1TO20 task. The kappa antagonist JDTic (10 mg/kg, ip) was administered to both groups of rats 2 h following the last training session (41). Starting 24-h following antagonist treatment, morphine and PPL-101 self-administration performance under the FR1TO20 schedule was monitored over seven daily 120-min sessions. Data are reported as number of infusions earned in daily 120-min sessions.

#### Assessment of Thermal Nociception of U-69,593 in Rats

##### Plantar Test

Thermal antinociceptive activity in rats was assessed by measuring hind paw withdrawal latency in response to radiant heat using a plantar test apparatus (Ugo Basile, Comerio, Italy) according to the method used by Hargreaves et al. (42). Briefly, each rat was placed into a compartment enclosure on a glass surface. A mobile heat source was then positioned under the plantar surface of the hind paw and activated with a light beam. A digital timer automatically recorded the response latency for paw withdrawal to the nearest 0.1 s. The mean withdrawal latency (seconds) for the left hind paw was determined from the average of three trials separated by a 10-min interval to prevent thermal sensitization.

To confirm that JDTic (10 mg/kg) was producing its effect on self-administration by blocking kappa receptors, we examined whether U-69,593-induced antinociception could be blocked using the rats that underwent self-administration testing. Paw withdrawal latency was measured in rats that were pretreated with JDTic or vehicle after completion of the self-administration experiments. One week after the JDTic administration, rats received an injection of U-69,593 (0.3 mg/kg, s.c.) or vehicle and were tested for paw withdrawal latency 15 min post-U-69,593 administration.

### Statistical Analyses

Antinociception in mice [% maximum potential effect (% MPE)] was quantified by the following formula: % MPE = 100 × [(test latency − baseline latency)/(15 − baseline latency)]. If the animal did not respond prior to the 15-s cutoff, a score of 100% was assigned. In initial assessment of PPL-101 and PPL-103, post-tail flick latency was analyzed using an ANOVA with drug treatment (PPL-101, PPL-103, and morphine) as a between-group variable and post-injection time as the dependent measure. PPL-101 time course data were analyzed using repeated measures ANOVAs with drug treatment (PPL-101, morphine, JDTic, or beta-FNA) as a between-group variable and post PPL-101-injection time (0.5, 1, 2, and 4 h) as the repeated measure followed by Bonferroni *post hoc* tests where appropriate. Global activity was analyzed using repeated measures ANOVAs with drug treatment (PPL-101, PPL-103, and morphine) as a between-group variable and injection day (first versus third) as a repeated measure. Significant interactions were further analyzed with one way ANOVAs and Bonferroni *post hoc* tests. To examine sensitization or tolerance to the development of global activity, following a significant overall ANOVA, *t*-tests were used to compare data following the third injection relative to the first. For the CPP test day data, a difference score was calculated as time spent in the drug-paired compartment minus time spent in the vehicle-paired compartment. Vehicle animals spent the same amount of time in both compartments such that the difference score is no different than 0 s. Difference scores were analyzed using ANOVAs, and significant effects were further analyzed with *post hoc* tests. CPP was evident if animals spent significantly more time in their drug-paired compartment, resulting in a positive difference score relative to control animals.

In rats, self-administration data (number of infusions, FR-1 schedule) were analyzed using repeated measures ANOVA with reinforcer (PPL-101, PPL-103, and morphine) as between-subject variables and “day” as a repeated measure. PR data, number of infusions, was analyzed using a one-way ANOVA with drug/dose (morphine, PPL-101) as the between-subject variable. Systemic effect of PPL-101 on morphine self-administration was analyzed by a one-way ANOVA that used treatment as a within-subject factor. Long-term effects of JDTic on morphine and PPL-101 self-administration were analyzed by comparing lever pressing before and after treatment using a one-way ANOVA. Paw withdrawal data were examined using a two-way ANOVA with drug pretreatment (vehicle or JDTic) and treatment (vehicle or U-69,593) as between-subject factors. *Post hoc* comparisons were conducted where appropriate.

For all experiments, the level of significance was set at *P* < 0.05.

## Results

### *In Vitro* Receptor and [^35^S]GTPγS Binding

Binding affinity and *in vitro* functional activity were determined at mu, kappa, and delta receptors for PPL-101, PPL-103, and several selective and non-selective reference compounds. As seen in Table 1, PPL-101 and PPL-103 have Ki of less than 5 nM at each receptor. Thus, the α-methyl-cyclopropylmethyl (α-methyl-CPM) moiety found in PPL-101 and PPL-103, as well as CPM found in cyclazocine and buprenorphine, induces high affinity for mu, kappa, and delta opioid receptors, unlike their N-CH_3_ counterparts, such as morphine, which have considerable selectivity for the mu receptors [Table 1; and see Ref. (16)]. The *in vitro* functional activity, as determined by [^35^S]GTPγS binding, is also affected by the N-substitution, as the α-methyl-CPM moiety induces a change in efficacy, so that PPL-101 and PPL-103 have low efficacy at mu and delta receptors and higher partial agonist activity at kappa receptors (Table 2). This is similar to what is found with cyclazocine (cycloproplymethyl-benzomorphan), which also has reduced efficacy at mu and higher efficacy at kappa relative to its N-CH_3_ analog, metazocine (43, 44). The ring structure also affects efficacy at the opioid receptors, since the oripavine buprenorphine, which also contains a CPM moiety, has reduced efficacy at mu receptors but no intrinsic activity at kappa.

**Table 1:** **Binding affinities Ki (nanomolars) of PPL-101 and PPL-103, compared with other prototypical agonists at the mu, kappa, and delta opioid receptors**.

	Receptor binding [Ki (nM) ± SEM]		
	Mu	Kappa	Delta
DAMGO	0.9 ± 0.1	305 ± 46	300 ± 59
DPDPE	503 ± 10	>10,000	1.6 ± 0.1
U-69,593	1,145 ± 335	0.3 ± 01	>10,000
Morphine	1.1 ± 0.1	47 ± 14.5	140 ± 1.5
Buprenorphine	1.5 ± 0.8	0.8 ± 0.1	4.5 ± 0.4
Cyclazocine	0.1 ± 0.01	0.1 ± 0.02	0.8 ± 0.05
PPL-101	0.35 ± 0.04	0.43 ± 0.1	4.0 ± 1.4
PPL-103	0.36 ± 0.11	2.47 ± 0.105	0.29 ± 0.03

*Data shown represent mean ± SEM for at least two experiments conducted in triplicate*.

**Table 2:** ***In vitro* functional activity of PPL-101 and PPL-103, compared with other prototypical agonists at the mu, kappa, and delta opioid receptors**.

	Mu		Kappa		Delta	
	EC_50_ (nM)	% Stim.	EC_50_ (nM)	% Stim.	EC_50_ (nM)	% Stim.
DAMGO	14 ± 5.3	100	4,400 ± 1,600	62 ± 21.0	>10,000	
DPDPE	>10,000		>10,000		1.3 ± 0.5	100
U-69,593	>10,000		26.1 ± 10.7	100	>10,000	
Morphine	16 ± 1.1	97 ± 1.05	575 ± 81	25 ± 2.0	412 ± 127	78 ± 0.9
Buprenorphine	2.3 ± 1.7	19 ± 05	>10,000		>10,000	
Cyclazocine	1.2 ± 0.07	33 ± 18	0.80 ± 0.2	80 ± 9	2.9 ± 1.9	82 ± 9
PPL-101	0.3 ± 0.1	12 ± 2.9	15 ± 2.5	63 ± 0.3	40 ± 6.3	22 ± 5.8
PPL-103	4.30 ± 2.13	22.6 ± 0.05	9.01 ± 2.64	39.8 ± 3.9	2.99 ± 0.92	41.7 ± 5.0

*Data shown represent mean ± SEM for at least two experiments conducted in triplicate*.

### Thermal Nociception of PPL-101 and PPL-103 in Mice

**Figure 2:**
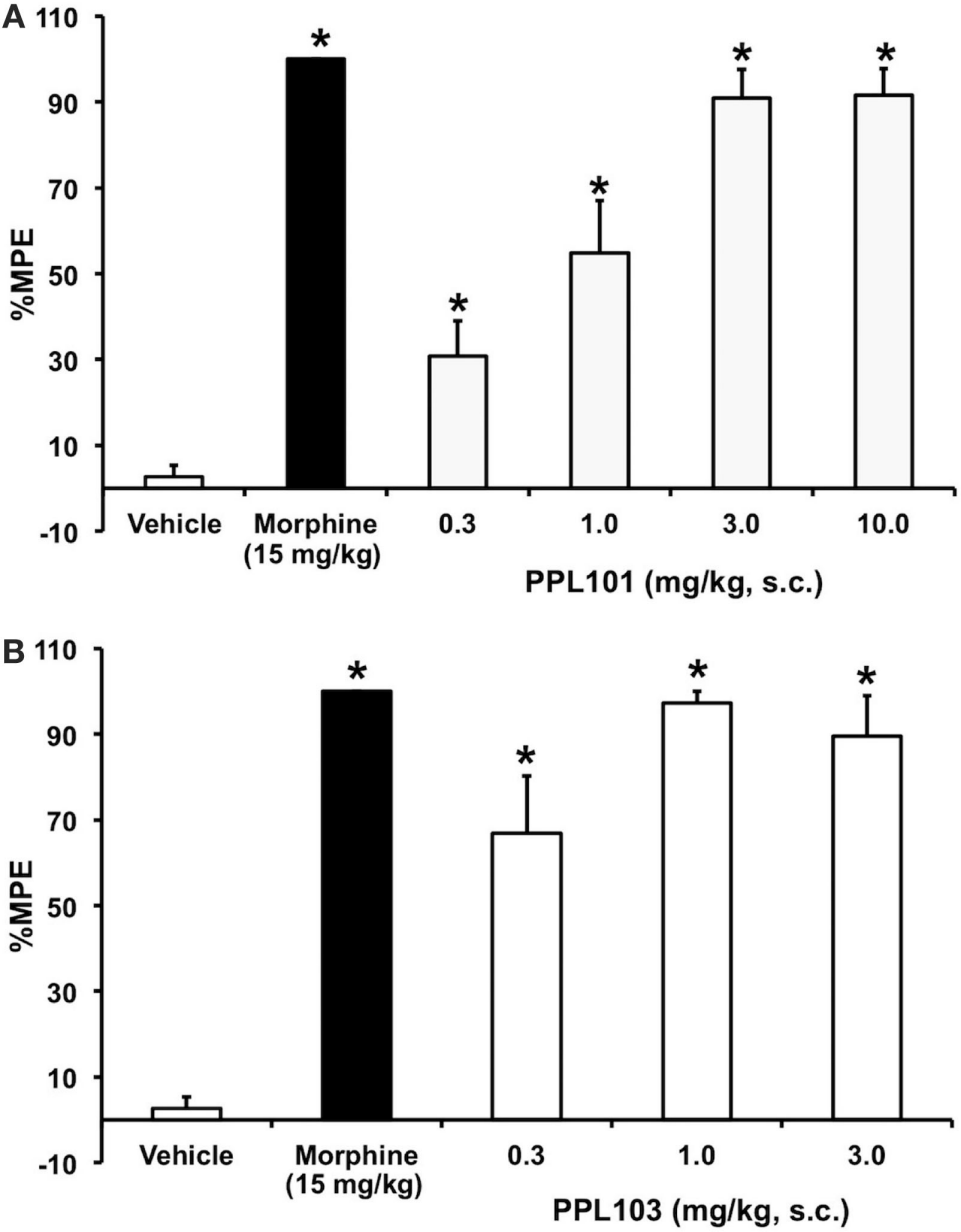
**Acute thermal antinociceptive effects of PPL-101 (A) and PPL-103 (B) alone using the tail flick assay in mice**. Data are mean % maximum potential effect (%MPE) (± SEM) 60-min post-injection. *, significant difference from vehicle control group (*P* < 0.05).

In initial assessments of thermal nociception, PPL-101 and PPL-103 exhibited significant antinociceptive activity measured by tail flick latency, as shown in Figure 2. The effects of PPL101 on tail flick latency 60-min post-injection are shown in Figure 2A. The overall ANOVA indicated that there was a significant effect of dose [*F*_(6,48)_ = 44.4, *P* < 0.05]. The positive control morphine produced the maximal effect in increase in tail flick relative to vehicle controls. The doses of 0.3–10 mg/kg PPL101 produced dose-dependent antinociception where there was a significant increase in %MPE relative to vehicle controls at all doses tested and maximal effects were observed with 3–10 mg/kg PPL-101.

PPL-103 also showed potent antinociception at all doses tested (0.3–3 mg/kg; Figure 2B). The overall ANOVA indicated that there was a significant effect of dose [*F*_(4,35)_ = 31.1, *P* < 0.05]. Maximal effects were observed following administration of the 1.0 and 3.0 mg/kg doses, whereas the 0.3 mg/kg dose produced a significantly reduced level of antinociception relative to the higher two doses.

**Figure 3:**
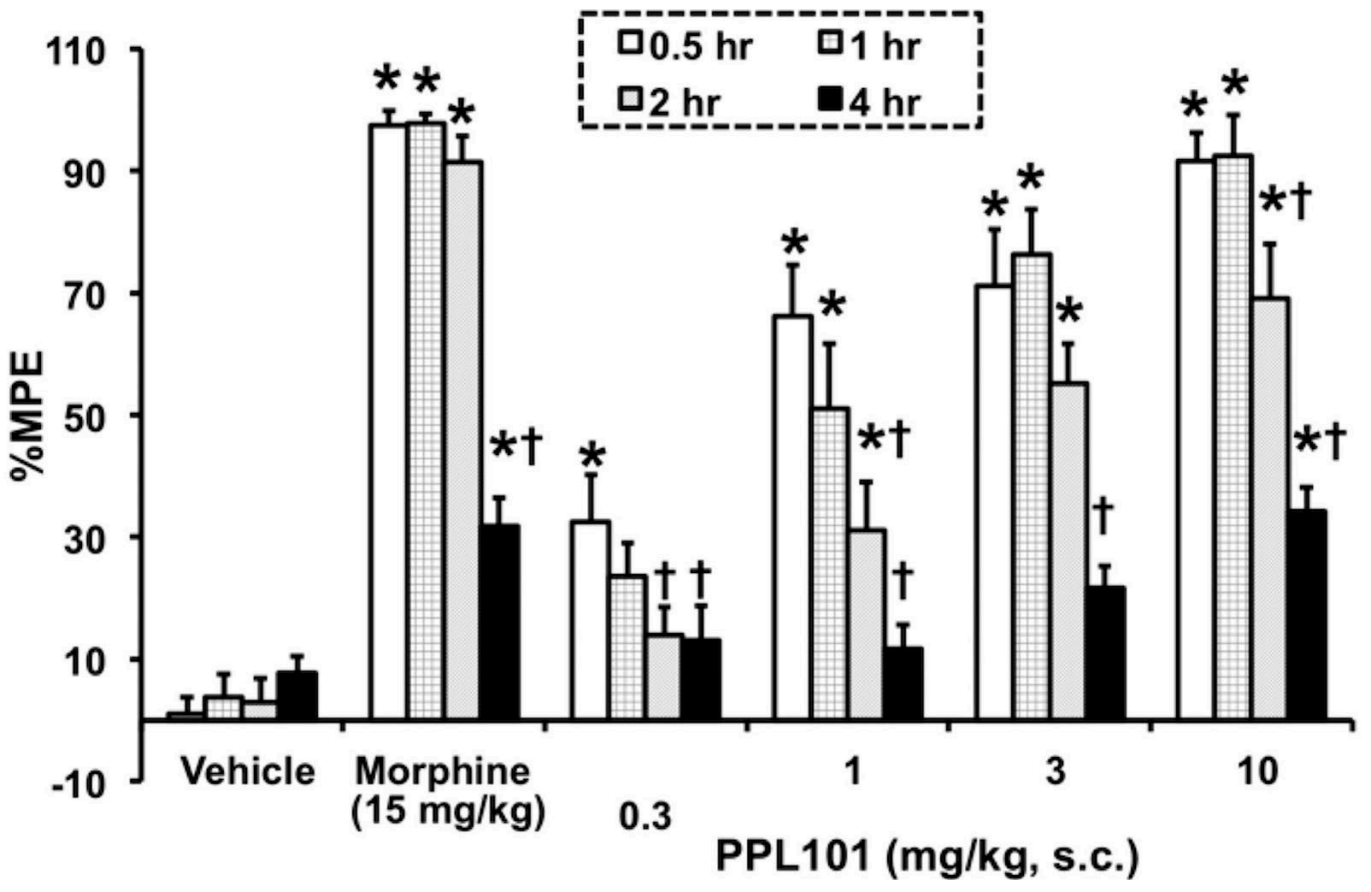
**Time course for acute thermal antinociceptive effect of PPL-101 (0.3–10 mg/kg, subcutaneous) alone in the tail flick assay in mice**. Data are mean % maximum potential effect (%MPE) (± SEM). *, significant difference from vehicle control group (*P* < 0.05). ^†^, significant difference from first test-point, 0.5 h (*P* < 0.05).

The time course for PPL-101 antinociception is shown in Figure 3. The overall ANOVA indicated that there was a significant dose by time interaction effect [*F*_(15,129)_ = 10.18; *P* < 0.0001]. The positive control morphine produced the anticipated increase in %MPE at all the time points compared to vehicle controls. Across time, %MPE produced by morphine was highest at the 0.5–2 h time points; however, there was a significant decrease in %MPE at the 4-h time point compared to the first time-point (0.5 h; *P* < 0.0001). PPL-101, at 0.3–10 mg/kg, produced a dose-dependent increase in %MPE that was significantly different from vehicle controls up to 4-h post-injection (Figure 3). Significant increases in %MPE were evident in all dosage groups of PPL-101 compared to vehicle controls at the first time-point (0.5 h; *P* < 0.001), whereas at the 1- and 2-h test points 1–10 mg/kg produced a significant increase in %MPE (*P* < 0.001). However, by the 4-h time point, only the highest dose of PPL-101 tested produced antinociceptive effects (*P* < 0.0001). The highest dose of PPL-101 produced similar levels of antinociception as the maximally effective dose of morphine tested and %MPE produced by 10 mg/kg PPL-101 was not significantly different than that produced by morphine at all time-points tested.

### Effect of JDTic and Beta-FNA on PPL-101-Induced Antinociception in Mice

**Figure 4:**
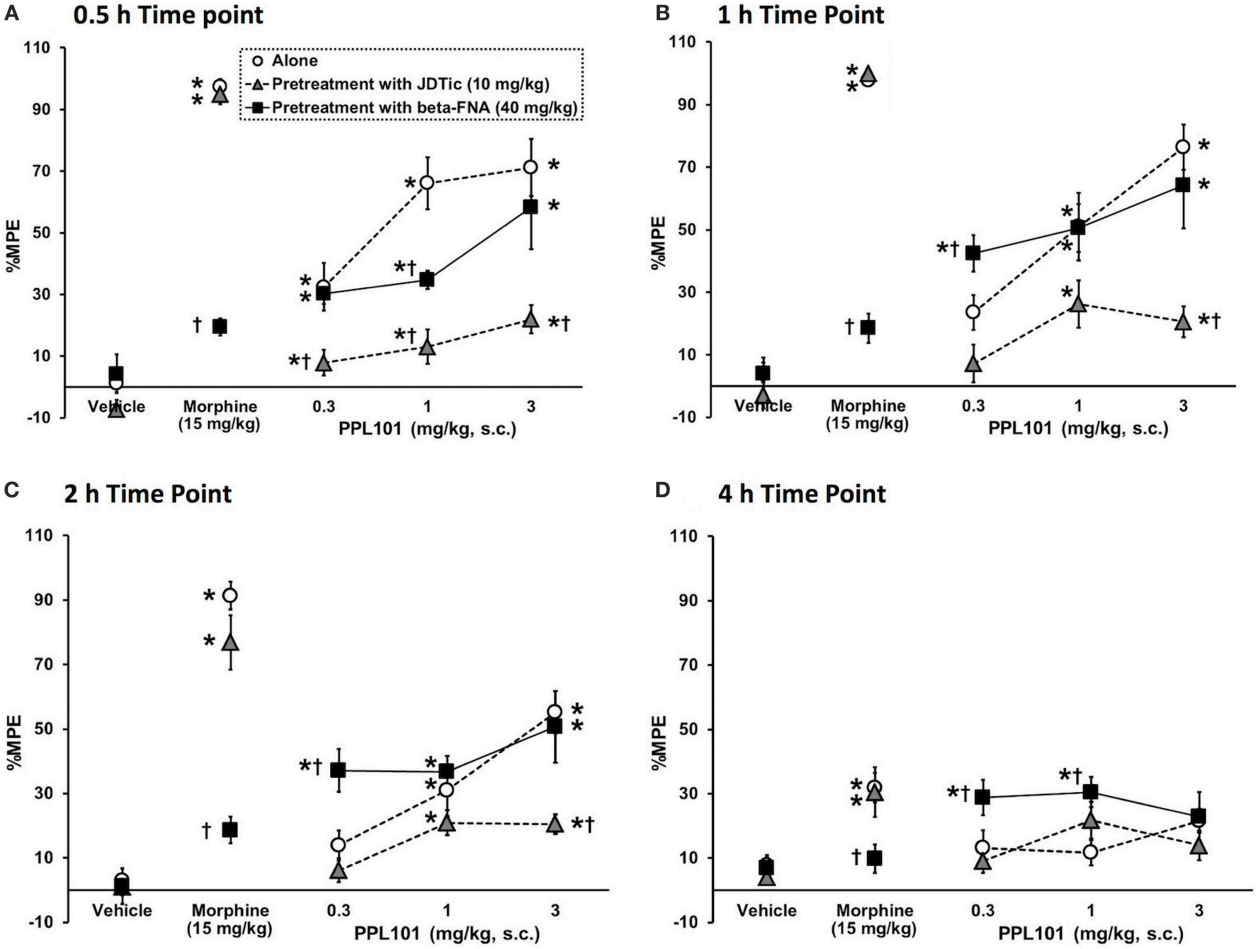
**The effect of kappa antagonist, JDTic [10 mg/kg, subcutaneous (s.c.)], and mu antagonist, beta-FNA (40 mg/kg, s.c.) on PPL-101-induced antinociception 0.5-h (A), 1-h (B), 2-h (C), and 4-h (D) post-PPL-101 injection in mice**. Data are mean % maximum potential effect (%MPE) (± SEM). *, significant difference from respective vehicle control groups (*P* < 0.05). ^†^, significant difference from PPL-101 alone (*P* < 0.05).

To determine the relative involvement of mu and kappa agonist activity in eliciting PPL-101-induced antinociception, animals were pre-treated with mu antagonist beta-FNA (40 mg/kg) or kappa antagonist JDTic (10 mg/kg; Figure 4) 24 h prior to PPL-101 injections and testing. The overall mixed three-way ANOVA indicated a significant interaction [*F*_(24,318)_ = 5.95; *P* < 0.0001]. As evident in the figure, JDTic (gray triangles) and beta-FNA (black square) when administered alone 24-h prior to a vehicle administration and testing have no effect on tail flick latency in mice. Morphine antinociception was completely inhibited by the mu opioid antagonist beta-FNA but was not altered by the kappa antagonist JDTic. On the other hand, PPL-101-induced antinociception was inhibited by JDTic and not beta-FNA. Although beta-FNA did not attenuate PPL-101-induced antinociception, it potentiated antinociception produced by 0.3 mg/kg PPL-101 at the 1- to 4-h post-PPL-101 injection, time-points during which the 0.3 mg/kg dose alone did not significantly produce antinociception (*P* < 0.05). Thus, it seems that in general, the kappa antagonist JDTic and not beta-FNA attenuated PPL-101-induced antinociception, whereas beta-FNA potentiated PPL-101 antinociception at the lowest dose.

### Reward Acquisition of PPL-101 and PPL-103 in Mice

**Figure 5:**
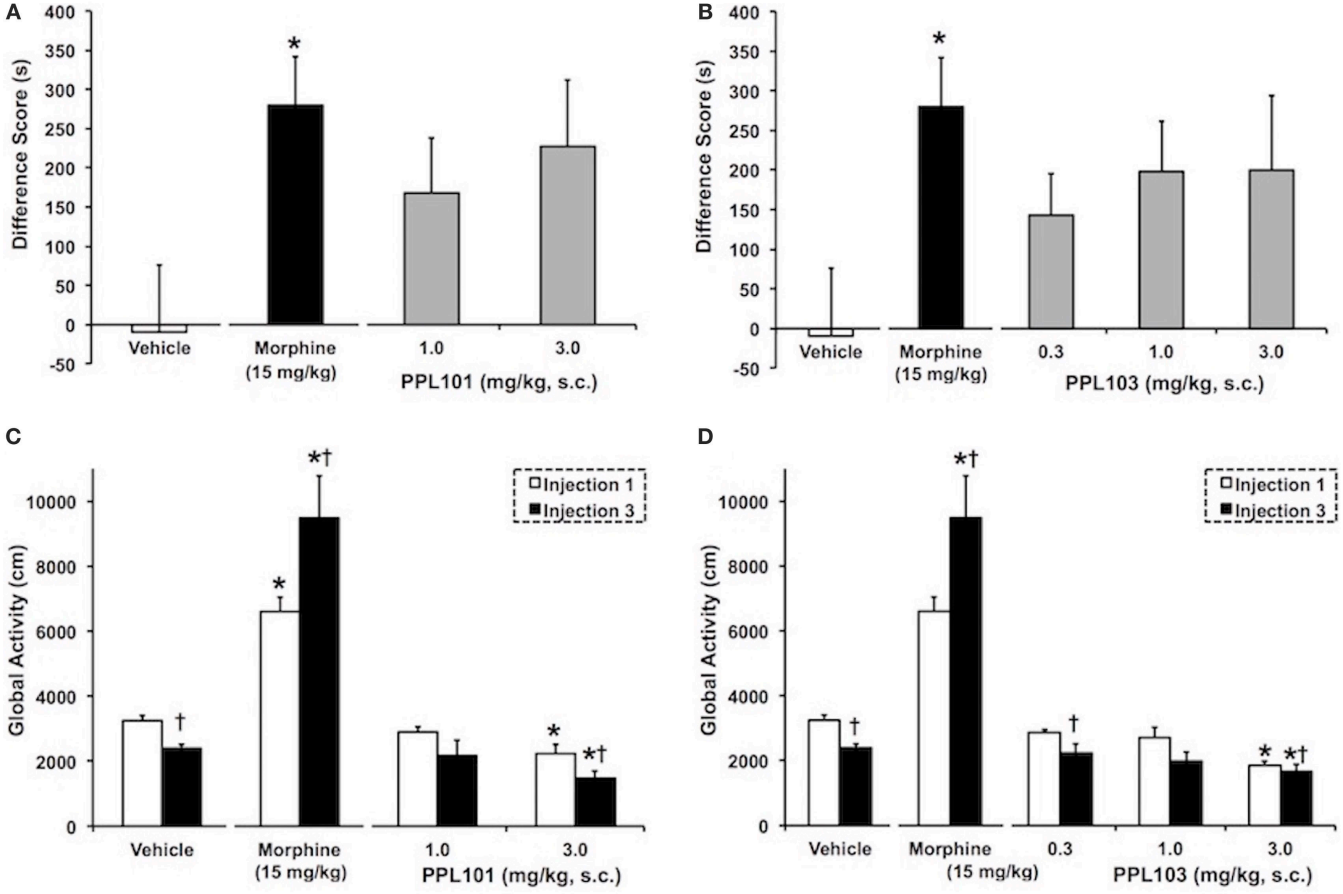
**The effect of PPL-101 [1–3 mg/kg, subcutaneous (s.c.)] and PPL-103 (0.3–3 mg/kg, s.c.) on conditioned place preference (CPP) (A,B) and on global activity (C,D) following the first (open bars) and third (black bars) drug injections in mice**. CPP data are mean (± SEM) difference score calculated as time spent in the drug-paired compartment minus time spent in the vehicle-paired compartment, whereas global activity data are activity (centimeters) following first and last drug injection. *, significant difference from vehicle control group (*P* < 0.05). ^†^, significant difference from first drug injection (*P* < 0.05).

The effect of PPL-101 and PPL-103 on acquisition of CPP is shown in Figures 5A,B. As expected, the group of animals that received 15 mg/kg morphine exhibited a significant CPP (*P* < 0.05) compared to vehicle controls. Although in Figures 5A,B PPL-101 and PPL-103 showed trends for CPP (*P* = 0.08 and 0.12 for PPL-101 and PPL-103, respectively), the *post hocs* did not reveal any significant difference between the groups that received PPL-101 or PPL-103 when compared to vehicle controls.

### Global Activity of PPL-101 and PPL-103 Following Acute and Repeated Administration in Mice

The effect of PPL-101 and PPL-103 on global activity during conditioning training, after the first and third drug injections, is shown in Figures 5C,D. The overall ANOVA indicated that there were significant dose by injection day interactions with PPL-101 [*F*_(3,25)_ = 7.9; *P* < 0.05] and PPL-103 [*F*_(4,34)_ = 7.8, *P* < 0.05]. As shown previously, morphine administration produced an increase in global activity after the first drug injection relative to vehicle controls (*P* < 0.05). Furthermore, sensitization of morphine-induced global activity was also evident as an increase in activity following the third drug injection relative to the first drug injection (*P* < 0.05). On the other hand, the 3.0 mg/kg dose of PPL-101 and PPL-103 produced decreases in global activity relative to vehicle controls following the first and third drug injections (*P* < 0.05). Additionally, 3 mg/kg PPL-101 produced a further decrease in global activity following the third drug injection relative to the first drug injection (*P* < 0.05).

### Reinforcing Properties of PPL-101 and PPL-103 in Rats

**Figure 6:**
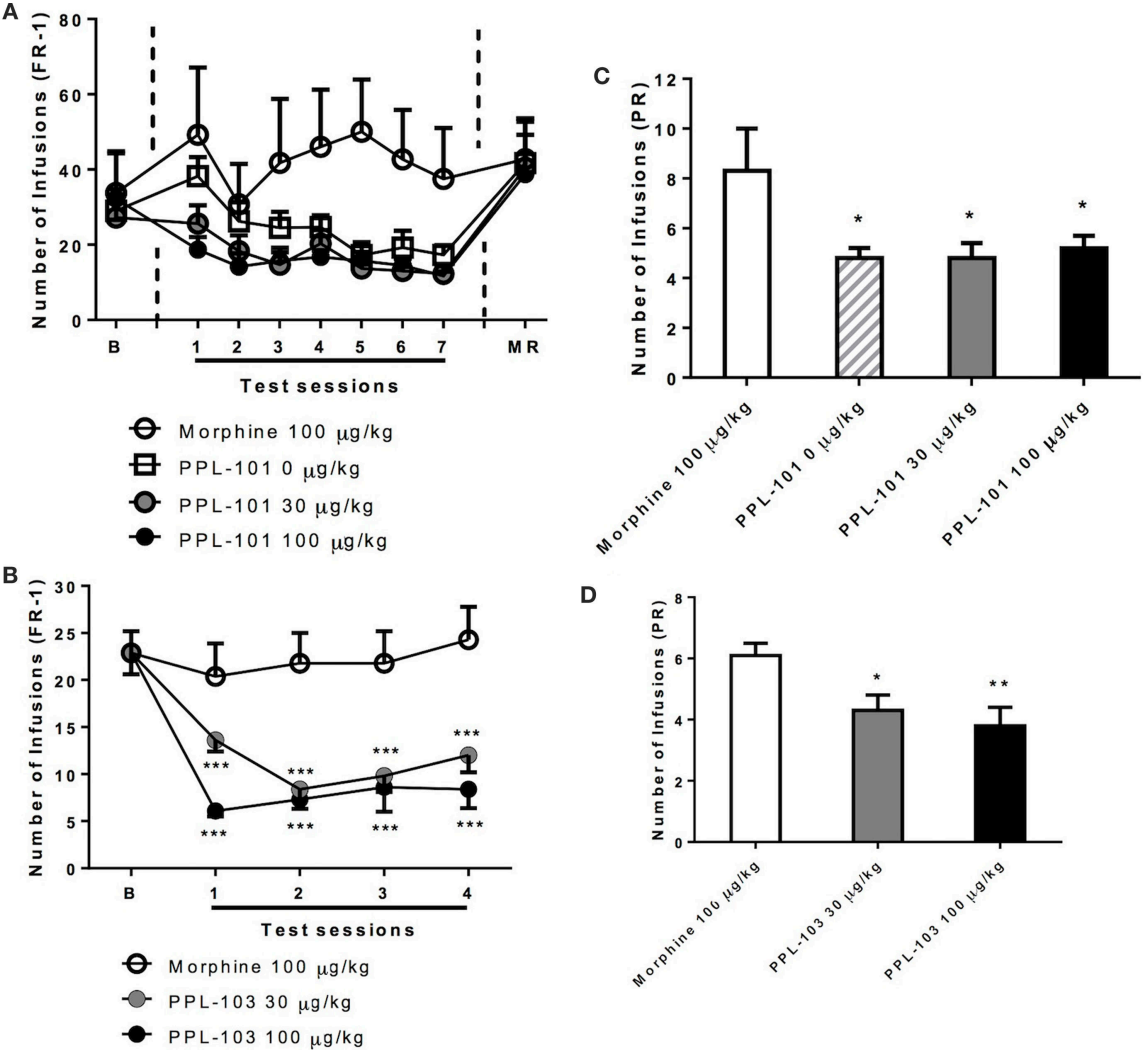
**Self-administration of PPL-101 (30 and 100 µg/kg/infusion) and PPL-103 (30 and 100 µg/kg/infusion) under fixed ratio 1 (FR-1) (A,B) and progressive ratio (PR) (C,D) schedules of reinforcement in rats**. For the PPL-101 experiment **(A,C)**, a between-subject design was used with four groups of *n* = 6. For the PPL-103 experiment **(B,D)**, a Latin Square within-subject design was used (*n* = 8). In both procedures, initial self-administration (baseline) was established with morphine (100 µg/kg/infusion). Data are mean (± SEM) number of infusions under both FR-1 and PR schedules. *P < 0.05, **P < 0.01, and ***P < 0.001 significant difference from morphine. B, baseline; MR, morphine re-acquisition.

Rats were initially trained to self-administer food pellets and then morphine sulfate at 100 µg/kg/infusion. Average responses on the morphine-associated lever over the last 3 days prior to PPL-101 self-administration were 29.0 ± 5.8, 27.2 ± 6.1, and 32.2 ± 12.6 (mean ± SEM). When PPL-101 (30 and 100 µg/kg/infusion) was substituted for morphine, rats exhibited a decrease in responding when compared to their counterparts that continued to self-administer morphine (Figure 6A). The overall ANOVA showed a significant main effect of “reinforcer” [*F*_(3,20)_ = 3.2, *P* < 0.05] that was not accompanied by a significant interaction “session × reinforcer” [*F*_(18,120)_ = 1.2, NS]. *Post hoc* analysis was therefore carried out on the collapsed factor of “reinforcer” (data for each PPL-101 dose group, morphine and vehicle groups averaged across the 7 days). This analysis indicated that both doses of PPL-101 were not self-administrated as compared to 100 µg/kg morphine (*P* < 0.05 for both doses, Figure 6A). Although by days 3–7 the 0.9% saline and PPL groups were very similar, the 0.9% saline replacement group only showed a trend toward a difference compared to the morphine group (*P* = 0.07).

Similarly, in a separate experiment with a new cohort of animals, when PPL-103 (30 and 100 µg/kg/infusion) was substituted for morphine using a within-subject Latin square design, the operant response under the FR-1 schedule clearly varied {reinforce × session interaction [*F*_(6,42)_ = 2.6, *P* < 0.05]}. *Post hoc* analysis showed that lever pressing for both doses of PPL-103 was markedly decreased as compared to responses for morphine across all test sessions (*P* < 0.001, Figure 6B).

When responding under the PR schedule (Figure 6C), similar changes in lever pressing were observed between groups that received PPL-101. These changes substantially paralleled those seen under the FR-1 schedule. Indeed, ANOVA revealed difference in the number of infusions [*F*_(3,20)_ = 3.0, *P* = 0.05]. *Post hoc* comparisons indicated that the number of infusions for vehicle and PPL-101 (30–100 µg/kg/infusion) were significantly decreased compared to the morphine group (*P* < 0.05). As with PPL-101, when responding under the PR schedule (Figure 6D), PPL-103 was self-administered to a lesser extent than morphine. ANOVA revealed difference in the number of infusions [*F*_(2,14)_ = 5.8, *P* < 0.05]. *Post hoc* comparisons indicated that the number of infusions for vehicle and PPL-103 (30–100 µg/kg/infusion) were significantly decreased compared to the morphine group (*P* < 0.05).

These data collectively suggest that at the doses tested, PPL-101 and PPL-103 are not self-administered by rats to the same extent as morphine and in fact were similar to the vehicle. This was further demonstrated by the evidence that all groups returned to high level of morphine-taking behavior when morphine replaced 0.9% saline, PPL-101, or PPL-103.

### Effect of PPL-101 on Morphine Self-administration in Rats

**Figure 7:**
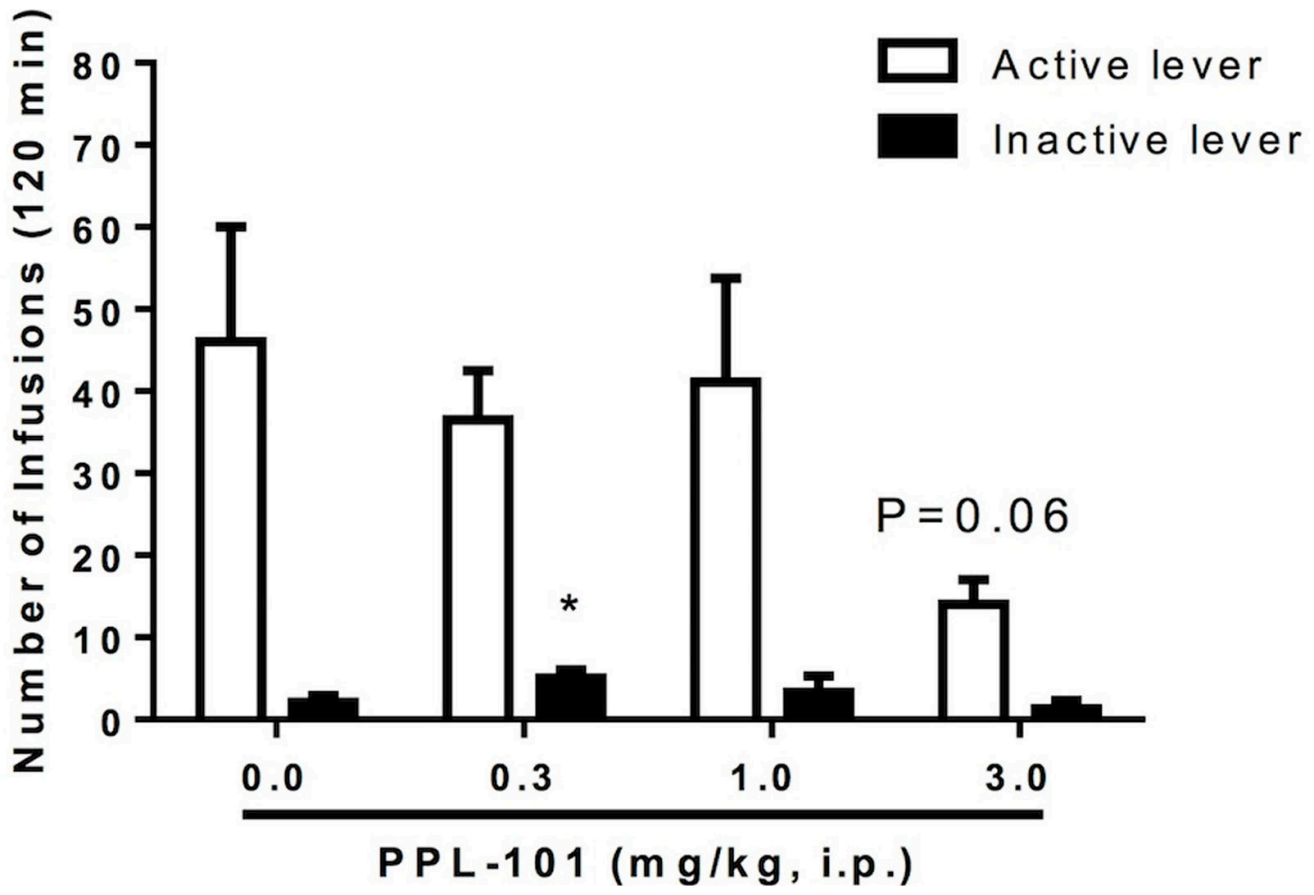
**Effect of PPL-101 [0.3–3.0 mg/kg, intraperitoneal (i.p.)] pretreatment on morphine (100 µg/kg/infusion) self-administration under an FR1TO20 schedule in rats**. Data are mean (± SEM) number of infusions during 120 min session. *P < 0.05 difference from vehicle treated group (PPL-101, 0.0 mg/kg).

Experiments were carried out to determine whether systemic pretreatment with PPL-101 would reduce morphine self-administration. PPL-101 (0.3–3.0 mg/kg; Figure 7) showed a marked trend to reduce operant responding for morphine. The overall ANOVA conducted on the morphine-paired lever revealed a main effect of PPL-101 treatment that was a very strong trend [*F*_(3,21)_ = 2.8; *P* = 0.06]. The same analysis conducted on the inactive lever led to an overall effect of treatment [*F*_(3,21)_ = 4.0; *P* < 0.05]. *Post hoc* comparisons revealed a significant increase in lever pressing in rats treated with PPL-101 0.3 mg/kg (*P* < 0.05), suggesting altered non-specific motor behavior only at this dose of PPL-101, while inactive lever responding did not change at higher doses.

### Effect of JDTic on Morphine and PPL-101 Self-administration

**Figure 8:**
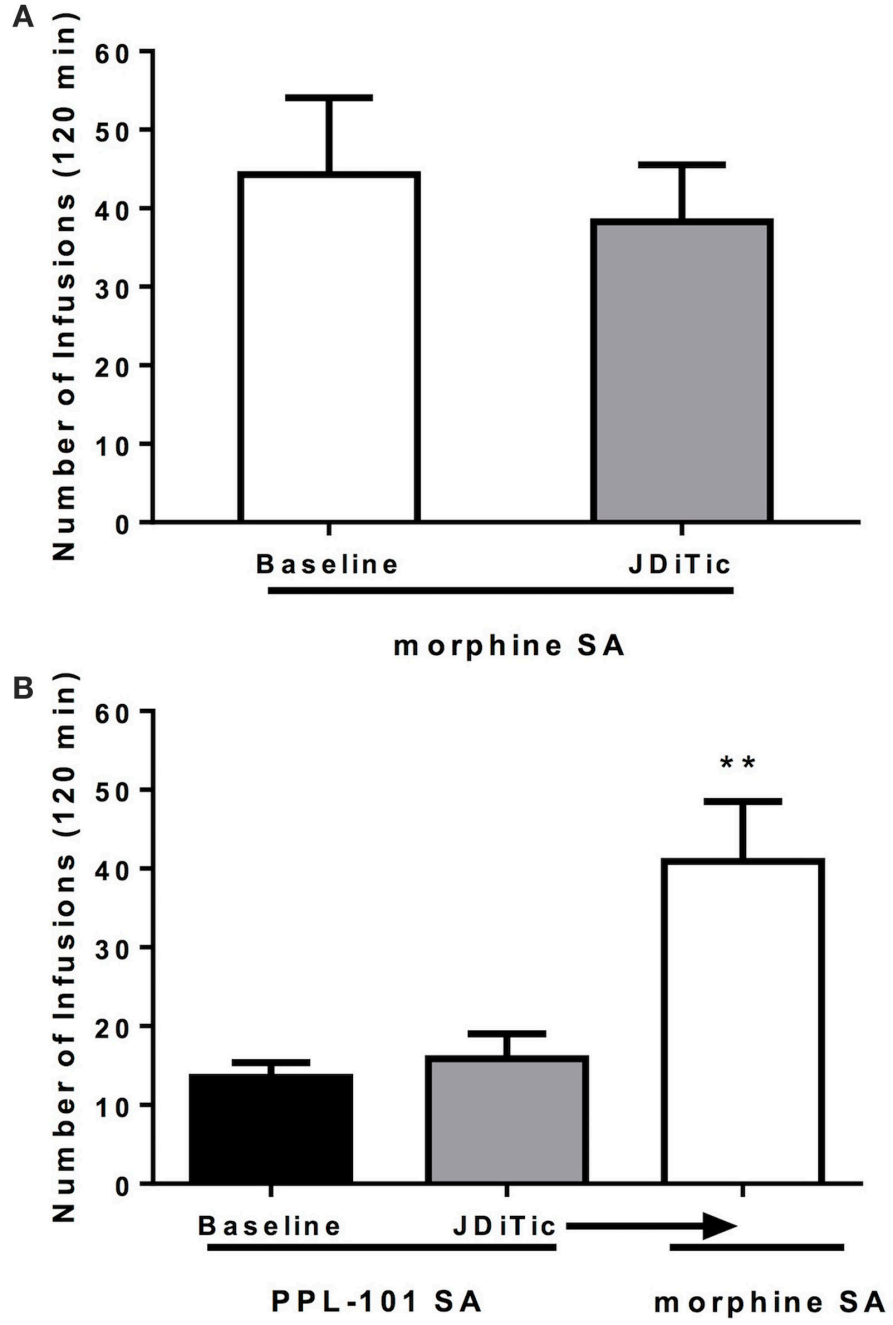
**Effect of kappa antagonist JDTic [10 mg/kg, intraperitoneal (i.p.)] on morphine (100 μg/kg/infusion) (A) and PPL-101 (100 μg/kg/infusion) (B) self-administration under an FR1TO20 schedule in rats**. JDTic was given 24 h prior to the first self-administration session. Data are the mean (± SEM) of the last 3 days of morphine SA prior to JDTic treatment compared to average lever pressing of the same rats treated with JDTic and self-administering morphine across the following seven sessions **(A)**, and baseline lever pressing for PPL-101 of the last three sessions versus the average lever pressing of JDTic-treated rats self-administering PPL-101 across 7 days **(B)**. **P < 0.01 difference from rats JDTic-treated and self-administering PPL-101.

Because PPL-101 was not self-administered, and partially activates both kappa and mu receptors, we examined whether this lack of drug-taking behavior was due to PPL-101 having very low mu-mediated activity or because kappa activity was high enough to diminish the mu-mediated reward. Rats were treated with the long-lasting kappa antagonist JDTic to block kappa activity during the self-administration sessions (Figure 8). Average lever pressing of JDTic-treated rats self-administering morphine (100 µg/kg/infusion) across seven daily sessions was compared to baseline lever pressing of the same rats across the last 3 days of morphine self-administration prior to JDTic treatment. ANOVA revealed no significant effect of JDTic on morphine self-administration [*F*_(1,4)_ = 1.5, n.s.]. Similarly, average lever pressing of JDTic-treated rats self-administering PPL-101 (100 µg/kg/infusion) across 7 days was greatly reduced compared to morphine self-administration and not different from baseline lever pressing of the same rats across the last 3 days of PPL-101 self-administration prior to JDTic treatment [*F*_(1,4)_ = 0.6, n.s.]. When morphine was substituted for PPL-101 in these JDTic-treated rats, the number of morphine infusions increased and was significantly higher compared to the number of infusions for PPL-101 in the same animals [*F*_(1,6)_ = 19.0, *P* < 0.01; Figure 8]. These data demonstrate that even in the presence of JDTic (and therefore the absence of kappa agonist activity) PPL-101 is still not self-administered suggesting that it is its low mu efficacy rather than kappa agonist activity that influences the reduced drug-reinforcing profile of PPL-101.

### Effect of JDTic on U-69,593-Induced Analgesia in Rats

**Figure 9:**
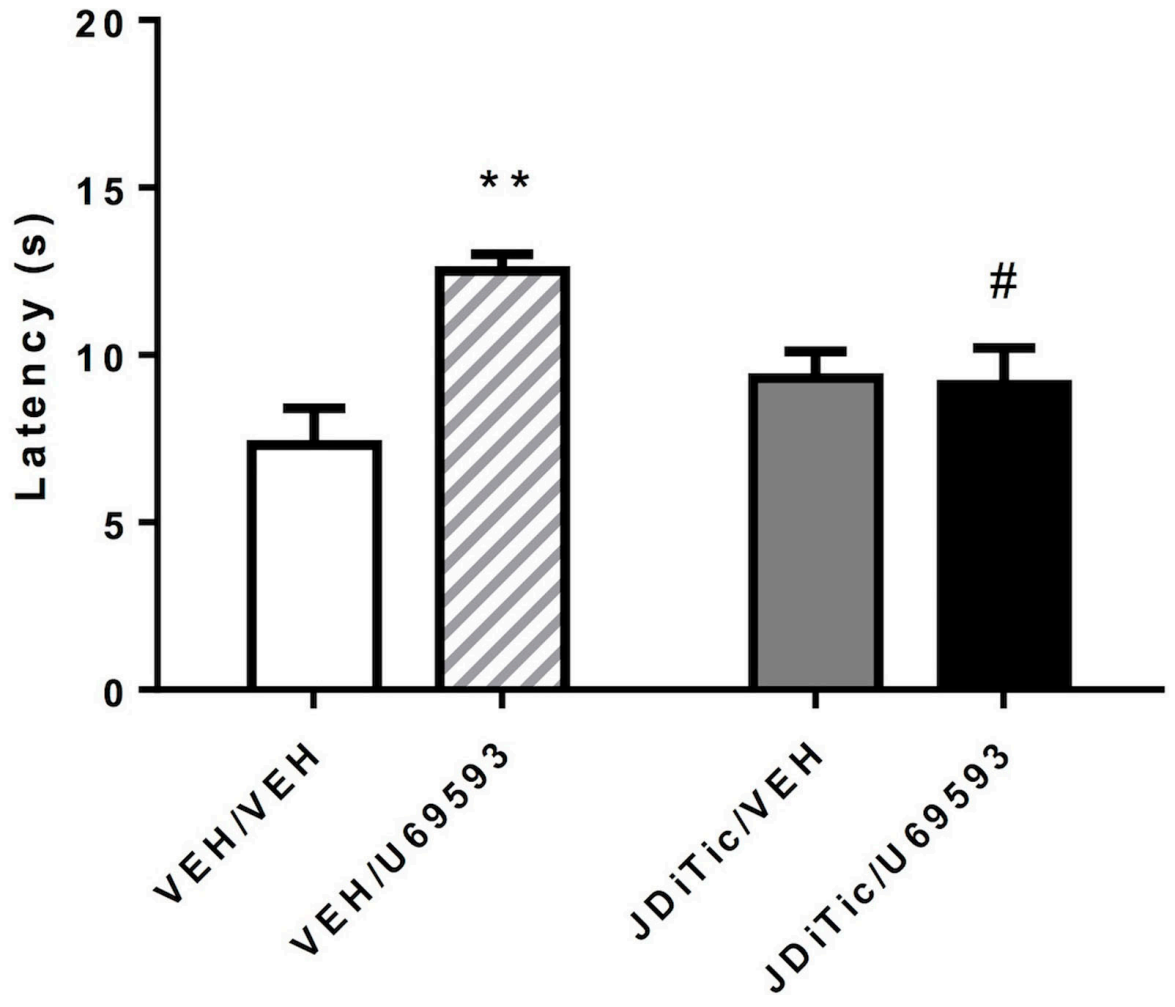
**U-69,593-induced antinociception using the plantar test in rats**. The kappa agonist U-69,593 (0.3 mg/kg) produced analgesia, an effect abolished by pretreatment with the kappa antagonist JDTic. Data are mean (± SEM) paw-withdrawal latency (seconds). **P < 0.01 difference from control group (VEH/VEH), ^#^*P* < 0.05 difference from U-69,593 treated group (VEH/U-69,593).

To confirm that JDTic (10 mg/kg) was functioning as a kappa antagonist during the self-administration experiments, we examined whether U-69,593-induced antinociception could be blocked using the rats that underwent self-administration testing. On plantar test, 0.3 mg/kg U-69,593 (i.p.) successfully induced antinociception [*F*_(1,16)_ = 7.4, *P* < 0.05], whereas the original single treatment with JDTic by itself did not [*F*_(1,16)_ = 0.7, NS]. ANOVA revealed “pre-treatment × treatment” interaction [*F*_(1,16)_ = 8.7, *P* < 0.01]. *Post hoc* pairwise comparisons, confirmed that the single dose of JDTic used (10 mg/kg) was able to block the antinociceptive effect U-69,593 (*P* < 0.01) and return paw withdrawal latencies to the level of controls (*P* < 0.05; Figure 9).

## Discussion

It has been demonstrated repeatedly that the N-substituent of several opioid backbones, including morphine, morphinan, and benzomorphan, greatly affects its affinity, selectivity, and efficacy at each opioid receptor. It has been known for many years that compared to morphine, the presence of a N-CPM moiety causes an increase in affinity at delta and kappa receptors, as well as a decrease in efficacy at mu receptors (15). Given the potential usefulness of opiates with N-substituent variations, such compounds have been carefully examined in both rodents and primates, leading to several compounds used in the clinic. In particular, the antagonist naltrexone and the partial agonist buprenorphine have a N-CPM moiety while nalbuphine and butorphanol both have N-cyclobutylmethyl substituents. Each of these clinically available compounds has relatively high affinity at each of the opioid receptors, with variations in the relative efficacy at each receptor, as do other N-CPM containing compounds such as kappa agonists cyclazocine and EKC (16, 20, 45, 46). None of these have an ideal profile as a non-addicting analgesic.

PPL-101, a morphine analog initially synthesized in 1979, and PPL-103, a recently synthesized morphinan version, have a CPM moiety as well as an additional methyl group attached to the α-carbon off the basic nitrogen. The presence of the alpha-methyl moiety restricts the rotation of the N-substituent and results in two diastereomers with different properties, the R-isomer having higher affinity and more potent analgesic activity than the S-isomer (26). PPL-101 and PPL-103 bind with high affinity to the mu and kappa receptors with a 10-fold lower binding affinity at delta opioid receptors. They both have low efficacy at mu and low to moderate efficacy at delta receptors with somewhat higher efficacy at kappa opioid receptors. Both compounds produced potent dose-dependent antinociception with PPL-103 being approximately 3 times more potent than PPL-101, making it roughly 10 times more potent than morphine in the mouse tail flick assay. Pretreatment with the long-lasting kappa antagonist JDTic, but not the irreversible mu antagonist beta-FNA, produced a downward shift of the PPL-101 dose–response curve indicating that the antinociceptive effects of PPL-101 were primarily due to kappa opioid receptor stimulation. This is interesting since even the prototypical kappa agonist EKC has sufficient mu receptor-mediated antinociceptive activity to be attenuated by beta-FNA (47). Despite the fact that PPL-101 is active through kappa receptors, its antinociceptive potency is five times that of the selective agonist U-50,488, and roughly three times that of morphine (48).

It is interesting to note that, although the mu receptor antagonist beta-FNA did not effectively decrease PPL-101 antinociception, nor did it have antinociceptive activity of its own when administered 24 h prior to the tail flick test, it was able to potentiate antinociception by the lowest dose of PPL-101. Partial agonist activity of beta-FNA at kappa receptors has been previously reported in smooth muscle preparations (49) and the acetic acid writhing test, though not in tail flick (50). Therefore, it is possible that some low level of kappa agonist activity of beta-FNA could potentiate antinociception produced by low doses of PPL-101.

In the present experiments, we also examined the potential rewarding and reinforcing effects of PPL-101 and PPL-103 using the CPP and self-administration paradigms in mice and rats, respectively. Typical kappa receptor agonists are powerfully dysphoric, produce a profound conditioned place aversion (51) and do not support self-administration in drug-naïve animals (52, 53). Furthermore, studies with the mu/kappa agonists such as butorphanol and nalbuphine, although initially reported to have lower dependence liability in humans, in animal models they produce mixed effects such that butorphanol produces CPP and supports self-administration, whereas nalbuphine only produces CPP at certain doses (54–56). PPL-101 or PPL-103 administration was neither aversive nor rewarding in mice, since animals did not spend significantly less or more time in their drug-paired compartment relative to vehicle controls, although surprisingly there was a trend toward CPP. This trend was not accompanied by the usual mu receptor agonist-mediated increase in locomotor activity, or by locomotor sensitization, a characteristic often considered as an indication of activation of the mesolimbic dopaminergic pathway. In fact, PPL-101 and PPL-103 both induced sedation at their highest dose, generally considered a kappa-mediated side effect (4). Furthermore, animals that were trained to self-administer morphine did not self-administer PPL-101 or PPL-103 in either FR or PR schedules. In order to determine whether PPL-101 was not self-administered because the mu activity was too low, or because the kappa activity counteracted mu reward, rats were injected with the long-lasting kappa antagonist JDTic prior to 1 week of PPL-101 and morphine self-administration. JDTic had no effect on rats that were self-administering either morphine or PPL-101. This suggests that, even in the absence of potential kappa receptor-mediated reduction of reinforcing properties, PPL-101 does not have sufficient mu activity to be self-administered.

Unlike the antinociceptive activity, subjective effects associated with PPL-101 and PPL-103 appear to suggest a mixed mu/kappa profile. Although the mu receptor component in PPL-101 seems to be very low, previous research in non-human primates have demonstrated that PPL-101 (then called NIH10497) was recognized as codeine and not EKC in a drug discrimination test, and substituted for morphine, thereby preventing withdrawal in morphine-dependent non-human primates (27, 28). Similarly, PPL-103 also substituted for morphine in a single-dose suppression test in non-human primates (Harris et al. unpublished observation). Thus, although the antinociceptive effects of PPL-101 and PPL-103 seemed to be orchestrated by kappa receptors, the effect of these compounds on reward, as demonstrated in the CPP paradigm, is distinctly not kappa like. Furthermore, despite apparent mu-mediated subjective effects, PPL-101 and PPL-103 are not reinforcing enough to be self-administered.

In conclusion, these results demonstrate that a mixed kappa/mu/delta compound, with varying partial agonist activity at each site, can produce interesting properties, where the final *in vivo* profile seems to be a function of the relative affinities and efficacies at the three opioid receptors. With PPL-101 and PPL-103, this profile includes potent kappa-mediated antinociceptive activity, an apparent lack of dysphoria, with no self-administration, and the ability to block morphine self-administration. Kappa agonists also are effective for reduction of itch (57), and kappa partial agonists have been postulated to be effective as a treatment for cocaine abuse (58). Therefore, a kappa agonist or partial agonist, without the accompanying dysphoria could prove useful for a number of ailments in humans.

## Ethics Statement

All animal care and experimental procedures complied with the Guidelines for the Care and Use of Mammals in Neuroscience and Behavioral Research [National Research Council (US) Committee on Guidelines for the Use of Animals in Neuroscience and Behavioral Research, 2003] and were approved by the IACUC of SRI International (Menlo Park, CA, USA; mouse antinociception and CPP studies) and the IACUC at the Torrey Pines Institute for Molecular Studies (Port Saint Lucie, FL, USA; rat self-administration).

## Author Contributions

Participated in research design: LT, TK, and AC. Conducted experiments: TK, AC, WP, and NT. Contributed New Reagents or analytic tools: WC and JL. Performed data analysis: TK and AC. Wrote or contributed to the writing of the manuscript: LT, TK, and AC.

## Conflict of Interest Statement

The authors declare that this study received partial funding from Phoenix PharmaLabs. Authors, LT, JL, and WC, are affiliated with Phoenix PharmaLabs. LT participated in research design and contributed to the writing of the manuscript. JL and WC synthesized compounds and made them available for the study. All other authors declare no conflict of interest.

## Abbreviations

% MPE, % maximum potential effect; JDTic, (3R)-7-hydroxy-N-[(1S)-1-[[(3R,4R)-4-(3-hydroxyphenyl)-3,4-dimethyl-1-piperidinyl]methyl]-2-methylpropyl]-1,2,3,4-tetrahydro-3-isoquinoline-carboxamide; PPL-101, α-methyl-cyclopropylmethyl-morphinan; PPL103, α-methyl-cyclopropylmethyl-morphine; beta-FNA, beta-funaltrexamine HCl; CPA, conditioned place aversion; CPP, conditioned place preference; DMEM, Dulbecco’s modified Eagle medium; FR, fixed ratio; i.p., intraperitoneal; i.v., intravenous; PR, progressive ratio; s.c., subcutaneous; SMART, spontaneous motor recording and tracking software system; TO, time out.
